# Risk factors associated with negative *in-vivo* diagnostic results in bovine tuberculosis-infected cattle in Spain

**DOI:** 10.1186/1746-6148-10-14

**Published:** 2014-01-13

**Authors:** Julio Álvarez, Andrés Perez, Sergio Marqués, Javier Bezos, Anna Grau, Maria Luisa de la Cruz, Beatriz Romero, Jose Luis Saez, Maria del Rosario Esquivel, Maria del Carmen Martínez, Olga Mínguez, Lucía de Juan, Lucas Domínguez

**Affiliations:** 1 Servicio de Microbiología, Instituto Ramón y Cajal de Investigación Sanitaria (IRYCIS), Crtra. Colmenar Viejo Km. 9.100, 28034 Madrid, Spain; 2 Centro de Vigilancia Sanitaria Veterinaria (VISAVET), Universidad Complutense Madrid, 28040 Madrid, Spain; 3 Center for Animal Disease Modeling and Surveillance (CADMS), Department of Medicine and Epidemiology, School of Veterinary Medicine, University of California, 95616 Davis, CA, USA; 4 CONICET/Facultad de Ciencias Veterinarias UNR, Casilda, Santa Fe, Argentina; 5 Dirección General de Producción Agropecuaria, Servicio de Sanidad Animal, Junta de Castilla y León, C/ Rigoberto Cortejoso 14, 47014 Valladolid, Spain; 6 Subdirección General de Sanidad e Higiene Animal y Trazabilidad, Ministerio de Agricultura, Alimentación y Medio Ambiente, 28071 Madrid, Spain; 7 Departamento de Sanidad Animal, Facultad de Veterinaria, Universidad Complutense de Madrid, 28040 Madrid, Spain

**Keywords:** Tuberculosis, Cattle, Diagnosis, Single tuberculin test, Interferon-gamma assay, Risk factors

## Abstract

**Background:**

Despite great effort and investment incurred over decades to control bovine tuberculosis (bTB), it is still one of the most important zoonotic diseases in many areas of the world. Test-and-slaughter strategies, the basis of most bTB eradication programs carried out worldwide, have demonstrated its usefulness in the control of the disease. However, in certain countries, eradication has not been achieved due in part to limitations of currently available diagnostic tests. In this study, results of *in-vivo* and *post-mortem* diagnostic tests performed on 3,614 animals from 152 bTB-infected cattle herds (beef, dairy, and bullfighting) detected in 2007–2010 in the region of Castilla y León, Spain, were analyzed to identify factors associated with positive bacteriological results in cattle that were non-reactors to the single intradermal tuberculin test, to the interferon-gamma (IFN-γ) assay, or to both tests applied in parallel (Test negative/Culture + animals, T-/C+). The association of individual factors (age, productive type, and number of herd-tests performed since the disclosure of the outbreak) with the bacteriology outcome (positive/negative) was analyzed using a mixed multivariate logistic regression model.

**Results:**

The proportion of non-reactors with a positive post-mortem result ranged from 24.3% in the case of the SIT test to 12.9% (IFN-γ with 0.05 threshold) and 11.9% (95% CI 9.9-11.4%) using both tests in parallel. Older (>4.5 years) and bullfighting cattle were associated with increased odds of confirmed bTB infection by bacteriology, whereas dairy cattle showed a significantly lower risk. Ancillary use of IFN-γ assay reduced the proportion of T-/C + animals in high risk groups.

**Conclusions:**

These results demonstrate the likelihood of positive bacteriological results in non-reactor cattle is influenced by individual epidemiological factors of tested animals. Increased surveillance on non-reactors with an increased probability of being false negative could be helpful to avoid bTB persistence, particularly in chronically infected herds. These findings may aid in the development of effective strategies for eradication of bTB in Spain.

## Background

Bovine tuberculosis (bTB) is a zoonotic disease of cattle primarily caused by *Mycobacterium bovis* and, to a lesser extent, by *M. caprae*. Despite decades of efforts to control and eradicate bTB, the disease persists in many developed countries [1]. Progress towards eradication has been variable, even within individual countries [2,3]. Many factors have been associated with persistence of this disease in a given region, including endemicity of the disease in the surrounding areas [4], herd breed and management systems [5,6], presence of wildlife reservoirs [7,8], and variable accuracy of diagnostic tests [9,10]. However, disease eradication at a farm level is considered achievable as long as the integrity of the herd (epidemiological unit) is maintained [11]. In this context, a diagnostic test that can accurately differentiate infected from non-infected animals at the individual level in a timely manner is crucial for disease eradication. Diagnostic test failures may result in false positive (lack of specificity) reactors or false negative (lack of sensitivity) non-reactors. False positives may have an important economic impact in the eradication programs due to the unnecessary slaughter of healthy animals and undermine the confidence of farmers in the program, while false negatives will maintain the infection in the herd. Thus, their detection is of paramount importance, especially as the overall prevalence of bTB decreases in a herd [9,12].

Single and comparative intradermal tuberculin (SIT and CIT respectively) tests, the most commonly used diagnostic techniques for detection of bTB-infected animals and herds, have been successfully applied for disease eradication in several regions of the world [13,14]. However, in certain countries and regions, those techniques have only been successful in decreasing bTB prevalence to a certain level [15], but without achieving eradication, probably due in part to the large variability observed in test sensitivity and specificity (especially at the individual level) [9,16]. In recent years, large-scale implementation of the interferon-gamma (IFN-γ) assay as an ancillary test has led to an increase of the individual diagnostic test sensitivity when used in infected herds [17]. However, impaired sensitivity has also been reported in certain situations, such as those with concurrent paratuberculosis infection [18], which further effects the interpretation of an individual test.

Both cervical SIT test (hereupon SIT) and IFN-γ assay are official diagnostic tests for bTB in the European Union, but they are currently being implemented using different protocols that may impact test sensitivity and specificity. One of the primary differences in the implementation of both tests across countries or regions depends on the test interpretation criteria, ‘severe’ or ‘standard’ in the case of the skin test (often a reflection of the expected prevalence in the herd), and in the cut-off point used in the IFN-γ, with up to eight different thresholds that are used in different member states [19].

In Spain, the national eradication program has been successful in achieving a sustained decrease of individual- and herd-level prevalence over the past 20 years (from 0.68 and 9.18% individual- and herd-prevalence, respectively, in 1991, down to 0.28 and 1.33%, respectively, in 2011) [20]. However, during the final stages of the eradication program, progress has slowed down, and a certain degree of variability in the sensitivity of the techniques (including SIT test and IFN-γ assay) has been described at the individual level under Spanish conditions [21].

This study was aimed at quantifying the number of non-reactors to the *in-vivo* diagnostic tests (SIT and IFN-γ tests) that subsequently produced culture positive results confirming *M. bovis* or *M. caprae* infection (false negative reactors) and the identification of individual (age) and herd (productive type, number of herd tests performed in the herd) factors associated with these false negative results in the *in-vivo* tests at the animal level. The potential impact of changing the interpretation or cut-off of the *in-vivo* tests was also assessed.

## Methods

### Study population

Every dairy (n = 29) and bullfighting (n = 28) herd that was detected as bTB-positive using the cervical SIT test and were subsequently confirmed as infected and also tested using the IFN-γ assay as an ancillary test in the Castilla y Leon (CyL) region in 2007–2010 was included in the study. Due to the large number of bTB-infected beef herds subjected to the same diagnostic scheme in 2007–2010 (n = 650), only a proportion of bTB-infected beef herds detected in 2007 and subjected to SIT and IFN-γ assay parallel testing (95 out of 150 so the proportion between dairy and bullfighting herds with beef herds was approximately 1:3) were randomly selected and included in the study.

Diagnostic tests were applied in parallel so animals were classified as reactors if positive to either the SIT or IFN-γ tests. According to the national eradication program reactors were culled and subjected to a post-mortem analysis (bacteriology, see below), but a number of non-reactor cattle, in which this study is focused, were also culled and subjected to post-mortem analysis for epidemiological reasons: this group included animals having one or more previous reactions close to (but below) the thresholds that were not considered reactors, possible anergic cattle (suspected paratuberculosis-infected cattle), progeny from infected animals and a subset of non-reactors sent to the slaughterhouse with positive cattle that were also randomly selected and sampled. Information on every culled animal that was subjected to post-mortem analysis (bacteriology) until the tuberculosis episode was resolved was recorded in a database, including animal identification number, farm of origin, date of birth, number of tests performed in the herd since the outbreak was disclosed, and quantitative (i.e., mm or optical density) and qualitative (positive/negative) results of the SIT test and IFN-γ assay respectively. Management and handling of the animals was performed according to the Spanish and European legislation (Council Directive 98/58/CE, RD348/2000). Information included in the study was exclusively derived from the work performed in the frame of the eradication programs, thus no experimental research on animals was performed.

### *In-vivo* diagnostic tests

At the first repetition after the disclosure test (the test in which the outbreak is first detected in the herd), performed in the following 2–6 months, all > 6 week old animals in the herd were tested using the cervical SIT test, performed by field veterinarians using bovine PPD according to the Spanish and European legislation (RD2611/1996, transposition of annex A of Council Directive 64/432/EEC) as described elsewhere [21]. Animals with a >2 mm increase in skin fold thickness and/or presence of clinical signs (oedema, exudation, necrosis, pain or inflammation of lymphatic ducts/lymph nodes) at the point of inoculation were defined as reactors (severe interpretation) and culled within the subsequent 14 days.

In addition, >6 month old animals were also subjected to IFN-γ testing. The IFN-γ assay was performed as described elsewhere [22]. All animals in which mean optical density (OD) of the sample stimulated with bovine PPD minus the mean OD of nil antigens was greater than 0.05 and greater than the OD of the avian PPD-stimulated sample were considered positive and culled within the following 14 days after the test was read.

### *Post-mortem* analyses

Animals culled due to positive reactions in the diagnostic tests and a proportion of non-reactors were slaughtered and sampled at the abattoir by the official veterinary services; samples from lung and retropharyngeal, bronchial and mediastinal lymph nodes were collected and submitted to the laboratory. Tissues were homogenized using a stomacher and decontaminated using the same volume of a N-acetyl-L-cysteine-sodium hydroxide (NALC-NaOH) solution (MycroPrep, Becton Dickinson, Franklin Lakes, USA), centrifuged for 20 min at 1300 g, resuspended in 1–3 ml. of phosphate buffer and inoculated onto one BACTEC MGIT tube supplemented with the antibiotic mixture polymyxin B, amphotericin B, nalidixic acid, trimethoprim and azlocillin (PANTA). Culture media were monitored by the BACTEC 960 instrument (Becton Dickinson) hourly during the entire 42 day incubation period. Identification of positive cultures was performed using a specific real time PCR for *M. tuberculosis* complex members targeting the p34 gene (Cultek, Madrid, Spain).

### Data analysis

Quantitative results of the *in-vivo* tests (skin fold thickness increase and OD readings) and bacteriology were recorded and linked with the individual identity of each animal. Animals were classified as positive/negative at each *in-vivo* test according to the official interpretations (SIT test: severe interpretation; IFN-γ assay: 0.05 threshold) as described previously. Furthermore, an additional interpretation, less restrictive, was also applied for the purpose of this study (evaluating the effect of a change in the official interpretation in place for each technique) in both tests [SIT test: animals were only positive if increase of skin fold thickness was ≥4 mm and/or any clinical signs were observed (standard interpretation); IFN-γ assay: use of a 0.1 cut-off point instead of 0.05]. Animals were classified as false negative for each alternative diagnostic test interpretation using bacteriology results (positive culture confirmed by PCR) as the gold standard (T-/C + animals).

Subsequently, the association between epidemiological and demographic features of the animals (age, productive type, and number of tests performed in the herd during the bTB outbreak before being culled) and the probability of *M. bovis* or *M. caprae* being cultured (i.e., of being T-/C+) was quantified in the animals that tested negative at each of the alternate test interpretations (‘severe’ or ’standard’ SIT test, IFN-γ assay using 0.05 or 0.1 as thresholds and interpretation of severe SIT test and 0.05 IFN-γ assay in parallel) generating five multivariate logistic regression models. For each of the five models, the subpopulation of animals analyzed included those animals that were negative to the *in-vivo* diagnostic test and the response variable in the model was whether *M. bovis* or *M. caprae* was cultured or not (positive/negative). Age was included as a categorical variable (i.e. quartiles) in the models and the first quartile was used as the reference category. Number of herd tests since disclosure of the outbreak was also categorized into four categories (one, two, three and four or more herd tests) with the first one being used as reference category. For productive type the category with the higher number of individuals (beef) was used as the reference category. To account for lack of independence in the observations, all models included herd as a random-effect. All three potential explanatory variables (age, productive type and number of herd tests performed in the herd) were tested in the multivariate models but only significant (p < 0.05) covariates were retained in the final model. Odds ratios and 95% CIs were estimated, and P-values ≤0.05 were considered significant. All possible two-way interaction terms between the significant explicative variables were evaluated. Goodness-of-fit of the final models was assessed using the Hosmer and Lemeshow test [23].

Statistical analyses were performed using R version 2.12.1 [24]. Mixed multivariate logistic regression models were analyzed using the lme4 package in R [25].

## Results

### Descriptive results

Over the 1-to-9 herd-tests per herd performed to control the bTB outbreaks, 3,614 animals (2,687 reactors at the SIT and/or the IFN-γ tests and 927 animals negative at both SIT and IFN-γ) from the 152 selected herds were culled and subjected to bacteriology (Table 1). The number of animals from each herd ranged from 1 to 234 (median = 14), 2 to 202 (median = 10) and 6 to 97 (median 31) in beef, dairy and bullfighting herds, respectively. The number of non-reactors differed depending on the diagnostic test applied and the cut-off in place (3,161 and 3,263 negative to the severe and standard SIT test respectively, 1,129 and 1,959 negative to the IFN-γ assay using 0.05 and 0.1 as cut-off points and 927 animals negative to both SIT and IFN-γ (Table 1). Median age was similar across groups, and 50-60% of the animals were beef cattle. The proportion of dairy and bullfighting cattle and the number of herd-tests performed in the herd since disclosure of the outbreak until the animal was culled varied depending on the test considered (Table 1). The proportion of non-reactors with a positive post-mortem was higher for the SIT test [24.3% (95% CI 22.8-25.8 using the severe interpretation and 24.1% (95% CI 22.7-25.7) with the standard interpretation] than for the IFN-γ (12.9% (95% CI 11.1-15.0) when the 0.05 threshold was used and 17.2% (95% CI 15.6-18.9) using the 0.1 cut-off). When both tests were used in parallel the proportion of culture-positive animals among non-reactors dropped to 11.9% (95% CI 9.9-11.4%) (Table 1).

**Table 1:** **Individual information of animals negative in the single intradermal tuberculin (SIT) test using severe and standard interpretations, the interferon-gamma (IFN-**γ**) assay using two cut-offs (0.05 and 0.1) and negative to both severe SIT and 0.05 IFN-**γ **tests that were subjected to bacteriology**

**Variable recorded**		**Negative in the SIT test**		**Negative in the IFN-γ assay**		**Negative in both tests**
**Interpretation/cut-off**		**Severe**	**Standard**	**0.05**	**0.1**	**Severe (SIT)/0.05 (IFN-γ)**
Number of animals		3161	3263	1129	1959	927
Median age (years) (IQR)		4.90 (1.73-8.69)	4.82 (1.72-8.65)	4.57 (1.83-8.12)	4.84 (1.85-5.83)	4.84 (1.97-8.47)
Productive type (%)	Beef	1776 (56.2)	1862 (57.1)	595 (52.7)	1183 (60.4)	456 (49.2)
	Dairy	512 (16.2)	522 (16.0)	324 (28.7)	370 (18.9)	286 (30.9)
	Bullfighting	873 (27.6)	879 (26.9)	210 (18.6)	406 (20.7)	185 (20.0)
Number of herd-tests before the animal was culled^a^ (%)	1	1268 (40.1)	1316 (40.3)	260 (23.0)	654 (33.4)	190 (20.5)
	2	850 (26.9)	868 (26.6)	306 (27.1)	535 (27.3)	253 (27.3)
	3	641 (20.3)	663 (20.3)	387 (34.3)	517 (26.4)	342 (36.9)
	> than 3	402 (12.7)	416 (12.7)	176 (15.6)	253 (12.9)	142 (15.3)
Bacteriology result (%)	Positive	768 (24.3)	788 (24.1)	146 (12.9)	337 (17.2)	110 (11.9)
	Negative	2393 (75.7)	2475 (75.9)	983 (87.1)	1622 (82.8)	817 (88.1)

^a^Since disclosure of the tuberculosis episode.

### Multivariate analysis

Each of the five different datasets of animals negative to the different diagnostic tests/interpretations was analyzed separately to evaluate the association between the odds of having a positive bacteriology result and the variables hypothesized to influence the odds.

**Figure 1:**
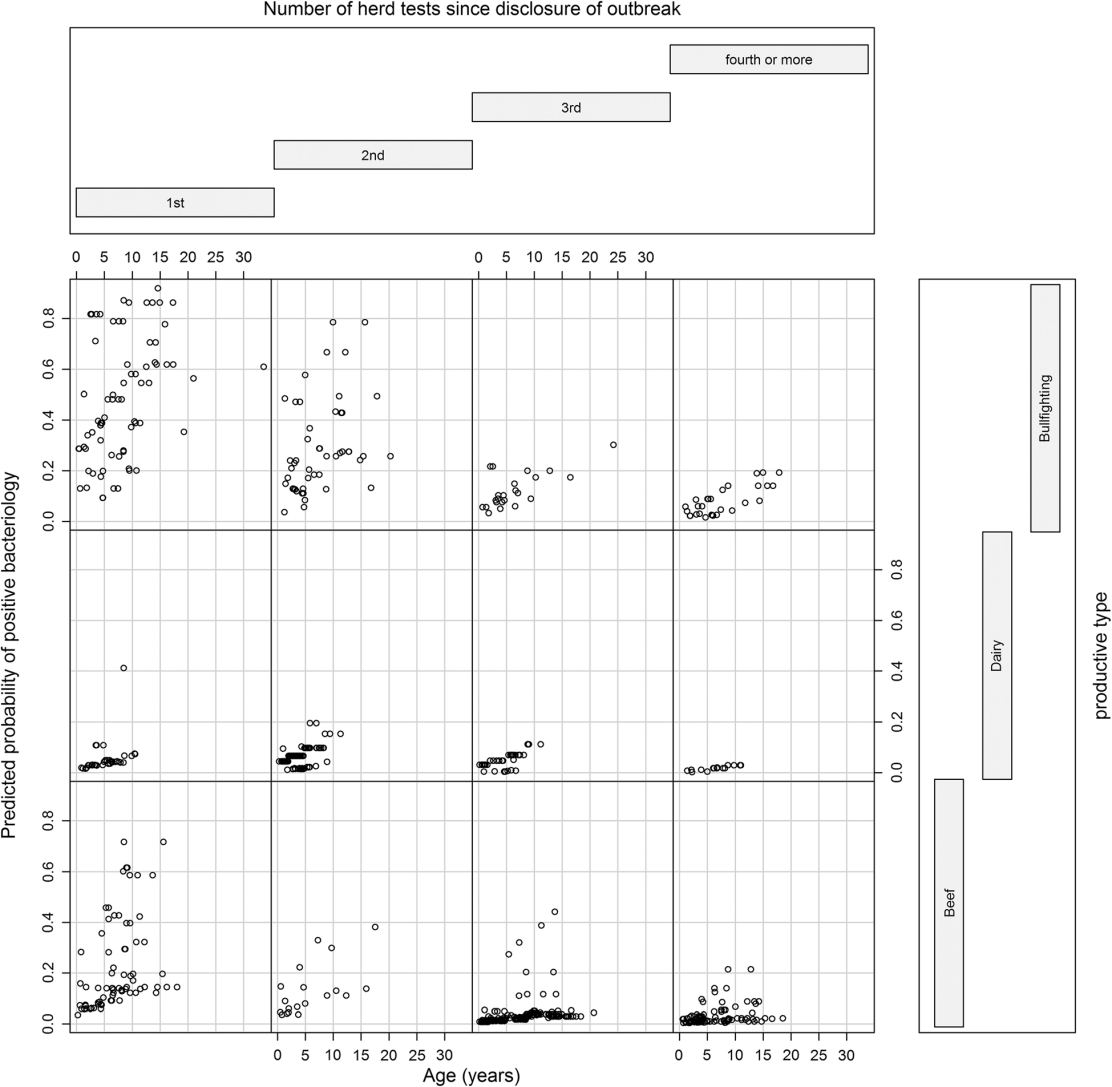
**Predicted probability of having a bTB positive result to bacteriology depending on the age, number of herd tests performed since disclosure of the outbreak in the herd, and productive type in cattle negative to the single intradermal tuberculin (SIT) test and the interferon-gamma (IFN-γ) assay from infected herds in Castilla y Leon, Spain.**

SIT negative animals: a higher proportion of culture-positive animals among non-reactors (using the severe interpretation) in older animals was observed (4.3% in the first quartil, 9.5% in the second, 12.4% in the third and 21.1% in the fourth). Statistical significance of this trend was confirmed by the multivariable analysis, as shown by the increasing OR in older animals (>4.9-8.7 years, OR = 3.7, 95% CI 2.2-6.3; >8.7 years, OR = 6.6, 95% CI = 3.9-11.1). Proportion of culture-positive animals was also higher for bullfighting cattle (1.9%, OR OR = 11.1, 95% CI 5.1-24.1) compared with beef (7.2%) and dairy (6.3%) animals, while decreased when the number of tests performed in the herd before the animal was culled increased (from 31.1% after the first herd-test to less than 5% after 3 or more herd tests) (Table 2). A significant interaction between age and productive type was detected, with a decreased odds of positive bacteriology results in older bullfighting cattle compared to beef cattle while no significant effect was observed in dairy animals (Table 2, Figure 1). Comparable results for the same covariates (age, productive type and number of tests) were obtained when the standard interpretation of the SIT test was applied in the selection of the negative animals (Table 2).

**Table 2:** **Results from the mixed logistic model of the probability of a positive bacteriology result for non-reactor animals at the single intradermal tuberculin (SIT) test, interferon-gamma (IFN-**γ**) assay and both tests used in parallel from infected herds from Castilla y Leon, Spain**

**Dataset (n)**	**Variable**	**Class (n)**	**N**	**Number of T-/C + animals (%)**	**OR**	**P**
Non-reactors if the severe SIT test and 0.05 IFN-γ assay are used in parallel: N = 927	Age	1st Q: 0.2-2	231	10 (4.3%)	1	NA
		2nd Q: >2-4.8	231	22 (9.5%)	1.52 (0.61-3.78)	0.36
		3rd Q: >4.8-8.5	233	29 (12.4%)	2.32 (0.97-5.53)	0.06
		4th Q: >8.5-33.6	232	49 (21.1%)	**3.89 (1.62-9.30)**	**0.002**
	Productive type	Beef	456	33 (7.2%)	1	NA
		Dairy	286	18 (6.3%)	0.26 (0.07-1.06)	0.06
		Bullfighting	185	59 (31.9%)	**3.64 (1.38-9.59)**	**0.009**
	Number of herd tests	1	190	59 (31.1%)	1	NA
		2	253	30 (11.9%)	0.58 (0.27-1.25)	0.17
		3	342	14 (4.1%)	**0.18 (0.08-0.43)**	**<0.001**
		4 or more	142	7 (4.9%)	**0.07 (0.02-0.21)**	**<0.001**
Non-reactors in the severe SIT test, N = 3,161	Age	1st Q: 0.2-1.7	790	88 (11.1%)	1	NA
		2nd Q: >1.7-4.9	790	154 (19.5%)	1.42 (0.75-2.66)	0.28
		3rd Q: >4.9-8.7	791	208 (26.3%)	**3.68 (2.16-6.26)**	**<0.001**
		4th Q: >8.7-33.6	790	318 (40.3%)	**6.59 (3.92-11.08)**	**<0.001**
	Productive type	Beef	1776	255 (14.4%)	1	NA
		Dairy	512	30 (5.9%)	0.27 (0.06-1-17)	0.08
		Bullfighting	873	483 (55.3%)	**11.1 (5.10-24.1)**	**<0.001**
	Interaction age*productive type	Bullfighting *1st Q	164	58 (35.4%)	1	NA
		Bullfighting *2nd Q	208	97 (46.6%)	1.28 (0.58-2.81)	0.56
		Bullfighting *3rd Q	205	85 (41.5%)	0.53 (0.26-1.09)	0.08
		Bullfighting *4th Q	296	102 (34.5%)	**0.50 (0.25-0.99)**	**0.04**
	Number of herd tests	1	1268	534 (42.1%)	1	NA
		2	850	134 (15.8%)	**0.33 (0.25-0.44)**	**<0.001**
		3	641	46 (7.2%)	**0.16 (0.11-0.24)**	**<0.001**
		4 or more	402	54 (13.4%)	**0.23 (0.15-0.34)**	**<0.001**
Non-reactors in the standard SIT test, N = 3,263	Age	1st Q: 0.2-1.7	816	89 (10.9%)	1	NA
		2nd Q: >1.7-4.8	815	157 (19.3%)	1.44 (0.78-2.64)	0.24
		3rd Q: >4.8-8.6	816	214 (26.2%)	**3.80 (2.26-6.37)**	**<0.001**
		4th Q: >8.6-33.6	816	328 (40.2%)	**6.39 (3.86-10.6)**	**<0.001**
	Productive type	Beef	1862	270 (14.5%)	1	NA
		Dairy	522	30 (5.7%)	0.25 (0.06-1.10)	0.07
		Bullfighting	879	488 (55.5%)	**10.6 (4.95-22.8)**	**<0.001**
	Interaction age*productive type	Bullfighting *1st Q	164	58 (35.4%)	1	NA
		Bullfighting *2nd Q	206	112 (54.4%)	1.30 (0.60-2.82)	0.51
		Bullfighting *3rd Q	209	121 (57.9%)	**0.50 (0.24-1.00)**	**0.05**
		Bullfighting *4th Q	300	197 (65.7%)	0.52 (0.26-1.02)	0.06
	Number of herd tests	1	1316	551 (41.9%)	1	NA
		2	868	136 (15.7%)	**0.33 (0.25-0.44)**	**<0.001**
		3	663	46 (6.9%)	**0.15 (0.10-0.23)**	**<0.001**
		4 or more	416	55 (13.2%)	**0.24 (0.16-0.35)**	**<0.001**
Non-reactors in the 0.05 IFN-γ assay: N = 1,129	Age	1st Q: 0.5-1.8	281	14 (5.0%)	1	NA
		2nd Q: >1.8-4.6	284	31 (10.9%)	1.45 (0.68-3.08)	0.34
		3rd Q: >4.6-8.1	281	41 (14.6%)	**2.20 (1.07-4.51)**	**0.03**
		4th Q: >8.1-33.6	283	60 (21.2%)	**2.86 (1.37-5.96)**	**0.005**
	Productive type	Beef	595	51 (8.6%)	1	NA
		Dairy	324	22 (6.8)	**0.25 (0.08-0.77)**	**0.02**
		Bullfighting	210	73 (34.8)	**3.13 (1.49-6.54)**	**0.002**
	Number of herd tests	1	260	80 (30.8%)	1	NA
		2	306	39 (12.7%)	0.55 (0.30-1.04)	0.06
		3	387	18 (4.7%)	**0.18 (0.09-0.36)**	**<0.001**
		4 or more	176	9 (5.1%)	**0.09 (0.04-0.23)**	0.059
Non-reactors in the 0.1 IFN-γ assay: N = 1,959	Age	1st Q: 0.5-1.9	488	30 (6.1%)	1	NA
		2nd Q: >1.9-4.8	492	66 (13.4%)	**1.76 (1.04-2.97)**	**0.03**
		3rd Q: >4.8-8.6	489	91 (18.6%)	**2.58 (1.57-4.24)**	**<0.001**
		4th Q: >8.6-33.6	490	150 (30.6%)	**4.98 (3.06-8.09)**	**<0.001**
	Productive type	Beef	1183	135 (11.4%)	1	NA
		Dairy	370	25 (6.8%)	0.38 (0.15-0.98)	**0.04**
		Bullfighting	406	177 (43.6%)	**5.70 (3.14-10.3)**	**<0.001**
	Number of herd tests	1	654	209 (32.0%)	1	NA
		2	535	70 (13.1%)	**0.37 (0.25-0.56)**	**<0.001**
		3	517	34 (6.6%)	**0.23 (0.14-0.38)**	**<0.001**
		4 or more	254	24 (9.5%)	**0.21 (0.12-0.36)**	**<0.001**

Significant values are indicated in bold.

*Indicates interaction between two variables.

NA: Non-applicable.

T-/C+: Animals negative in the *in-vivo* diagnostic test and positive in bacteriology.

IFN-γ-negative animals: when the 0.05 cut-off point was used to denote a positive case the proportion of culture-positive cattle increased in older animals (from 5% in the first quartile to 21.2% in the fourth), as reflected by the increasing OR as the age increases (>4.6-8.1 years, OR = 2.2, 95% CI 1.1-4.5; >8.1-33.6 years, OR = 2.86, 95% CI = 1.4-6.0).Bullfighting cattle had also a higher proportion of culture-positive cattle (34.8%. OR = 3.1, 95% CI 1.5-6.5) compared to beef (8.6% culture-positive cattle), whereas lower odds were estimated for dairy cattle (6.8% culture-positive, OR = 0.25, 95% CI 0.08-0.77) and in animals culled after the 2nd herd-test (with less than 6% of culture-positive animals in those groups) (Table 2). Again, comparable results were identified when the 0.1 threshold was used for the definition of a positive case (Table 2). However, the proportion of T-/C + animals in the groups with significantly larger ORs in each model was higher when the 0.1 cut-off was used as compared with the 0.05 cut-off (bullfighting cattle: 43.6% (95% CI 38.9-48.5) vs. 34.8% (95% CI 28.7-41.4) T-/C + cattle using 0.1 and 0.05 cut-offs respectively; older (4^th^Q) cattle: 30.6% (95% CI 26.7-34.8) vs. 21.2 (95% CI 16.8-26.3) T-/C + cattle using 0.1 and 0.05 cut-offs respectively), as well as the proportion of T-/C + cattle remaining in the herd after several herd tests (10.5% (95% CI 7.1-15.1) vs. 5.1% (95% CI 2.7-9.4)). In this case, an interaction between the number of herd tests before slaughter of the animal and productive type could not be assessed because after its inclusion the model did not converge.

Negative animals to both tests in parallel: the highest proportion of T-/C + animals was observed in older animals (21.1% in cattle > 8.5 years compared with 4.3% in those <2 years, OR = 3.9, 95% CI 1.6-9.3) and bullfighting cattle (31.9% compared to 7.2% in beef cattle, OR = 3.6, 95% CI 1.4-9.6) negative to both tests (Table 2, Figure 1). Lower odds were marginally significantly associated with dairy cattle (6.3% T-/C + animals, OR = 0.3, 95% CI 0.1-1.1) as compared to beef cattle. Odds were also significantly lower after more than two herd-tests after disclosure of the outbreak (Table 2).

All five models had good fit to the data (Hosmer and Lemeshow test, P > 0.05).

## Discussion

Unsatisfactory accuracy of diagnostic tests has been largely identified as one of the most critical factors hindering the success of bTB eradication programs [10]. Currently available diagnostic techniques may lack sensitivity, particularly in recently infected animals [9], which may represent the largest proportion of infected cattle remaining in herds subjected to frequent test-and-cull schemes. Thus, identification of infected cattle with the highest risk of being undetected may be of most use when evaluating herds in which infection is not cleared after a number of herd-tests. In this study our aims were to detect factors associated with higher odds of diagnostic failure (negative *in-vivo* results but positive bacteriology).

In the SIT-negative cattle age had a significant effect on the odds of having a positive culture (Table 2), although this effect was less pronounced in bullfighting animals compared to other productive types. However, among non-reactors in infected herds, animals included in the third (>4.9-8.7 years, 26.3% of T-/C + cattle) and fourth (>8.7-33.6 years, 40.3% of T-/C + cattle) age quartiles had a greater odds of a positive culture than younger animals (up to an OR = 6.6, 95% CI 3.9-11.1 for the fourth quartile). Age has been previously associated with increased risk of disease [26] and skin test positivity [27], but our results suggest that age is also associated with disease in non-reactors. Therefore, in chronically infected herds in which the SIT test is the only diagnostic test in place, older (>8 years) non-reactors should be monitored and removed from the herd when possible in order to decrease the chances of allowing infected animals to remain in the population. Lack of reliability of diagnosis in infected older animals may also be related to a state of anergy in chronically infected herds, in which a number of animals could be in advanced stages of the disease, likely due to a previous diagnostic failure. SIT-negative bullfighting cattle had also significantly higher odds of being positive to the bacteriology (Table 2) compared to beef and dairy SIT-negative animals (OR = 11, 95% CI 5–24). These results are in agreement with the poor individual test sensitivity (10.6% using the comparative skin test) previously reported in bullfighting cattle from the Camargue area [10]. Performing the SIT test in bullfighting cattle is challenging due to the dangerous temperament of the animals, which may explain, at least in part, the poor performance of the test. In addition, a breed-specific effect on the variability of the reaction to the SIT test cannot be ruled-out [26]. The increased risk of finding culture-positive cattle among bullfighting SIT-negative cattle (reflected in the 55% of culture-positive animals found in our dataset compared with 14.4 and 5.9% in beef and dairy cattle respectively) strongly suggests the need for using a complementary testing technique to decrease the number of false negative reactors that could be expected in bullfighting cattle. We also found that as the number of herd tests performed in the herd using SIT test and IFN-γ assay increased, the risk of finding positive culture results in non-reactors decreased (with less than 5% of T-/C + cattle after the fourth use of both tests in parallel), what may be due to a lower infection prevalence persisting in the herd. As expected, the probability of finding a positive culture also increased in SIT-negative animals that were positive to the IFN-γ assay (OR = 2.02, 95% CI 1.50-2.74, data not shown). The use of the standard instead of the severe interpretation in the analysis showed a similar trend, identifying the same risk factors with similar regression coefficients (Table 2).

Regarding IFN-γ assay negative animals, an age-related trend was observed similar to that described for SIT-negative animals, with older cattle being at significantly higher risk compared to young cattle (>4.6-8.1 years: OR = 2.2, 95% CI 1.1-4.5; >8.1-33.6 years: OR = 2.9, 95% CI 1.4-6.0). However, this effect was of lower magnitude than that described in the SIT-negative cattle population [overall proportion of T-/C + in the 4th Q was 21.2% (95% CI 16.8-26.3) in IFN-γ negative (0.05 threshold) cattle and 40.3% (95% CI 36.9-43.7) in SIT-negative (severe interpretation) cattle] suggesting that the performance of the IFN-γ assay was better for detection of older bTB-infected animals. Bullfighting cattle also had a high risk of having positive culture results (Table 2), but again the overall proportion of T-/C + bullfighting cattle in this group was lower than that observed in the SIT-negative cattle using the severe interpretation (34.8%, 95% CI 28.7-41-4 vs. 55.3%, 95% CI 52.0-58.6) thus indicating a better performance of the IFN-γ assay in this productive type. Interestingly, significant differences were observed between beef and dairy animals, with non-reactors from beef herds having 4 times higher risk of positive isolation than dairy cattle (Table 2). IFN-γ assay is a standardized laboratory technique that should not be affected by differences in breed-derived handling issues (if the sample is collected correctly). Therefore, differences associated with the productive type may be due to different stages of infection in each productive type or to differences in the immune responses observed in infected beef, dairy and bullfighting cattle. A significantly lower probability (5.6 times lower, 95% CI 2.8-11.3) of culture-confirmed infection was only observed in negative IFN-γ animals after the second herd test, when the observed proportion of culture-positive cattle was below 6% (Table 2). These results indicate that, to significantly increase the accuracy of negative results (i.e., the negative predictive value) the test should be performed at least twice in infected herds. In the population of non-reactors obtained after changing the threshold from 0.05 to 0.1 the proportion of T-/C + in bullfighting cattle and animals in older (>8.1 years) age classes was larger (from 34.8% of T-/C + bullfighting cattle to 43.6% if the threshold was moved from 0.05 to 0.1, and from 21.2% in fourth-quartile age animals – 0.05 threshold – to 30.6% using 0.1, Table 2) thus suggesting that the use of the 0.1 cut-off value would have a greater negative impact on the performance of the test in these groups with greater odds.

Test results were interpreted according to the two official interpretations of the SIT test and two possible cut-off points in the IFN-γ assay (the one currently in place in Spain and an alternate threshold that would provide higher specificity but lower sensitivity) [20] in an attempt to assess the impact of changing the interpretation/threshold on the risk of *in-vivo* diagnostic test failure. All the variables evaluated were significantly associated with increased risks of finding T-/C + animals regardless interpretation/cut-off, but higher proportions of T-/C + were found when the alternative interpretations (standard and 0.1) were used. This suggests that the use of diagnostic tests with higher specificity in infected settings may increase the probability of leaving infected cattle belonging to higher risk groups (bullfighting and older animals) even when more than 3 herd-tests were performed after disclosure of an outbreak. When both techniques were applied in parallel according to the Spanish legislation for bTB-confirmed infected herds (SIT: severe interpretation and IFN-γ: 0.05 threshold) the same associations detected for the IFN-γ assay remained significant and similar percentages of T-/C + cattle were observed for each category of the analyzed variables (Table 2). With this diagnostic strategy, the risk would be highest in bullfighting and older (>8.5 years) cattle, and at least two rounds of testing in parallel would be required to show a significant decrease in the odds of having a positive culture in non-reactors (Figure 1). All models fitted the data well as suggested by results of the Hosmer and Lemeshow tests.

Non-reactors at both tests were culled due to a variety of reasons that may differ from herd to herd, which may create a selection bias of animals included in this dataset. For example, a larger proportion of anergic cattle may be suspected in bullfighting-infected herds due to the higher likelihood of finding bTB-infected animals in advanced stages of the disease (associated with a higher prevalence of disease in this productive type in Spain). However, the same criteria were applied for selection of non-reactors in all herds regardless of productive type or number of herd tests after disclosure of the outbreak, and therefore results here can be considered as an indicator of the potential risk profile associated with non-reactors in each productive type. Even if more false negative animals may have remained in the herds selected in the study, results here can be considered representative of at least a proportion of the bTB infected-nonreactor population existing in each cattle herd depending on their previous bTB history (that could not be evaluated in this study) or other characteristics, such as productive type. The proportion of T-/C + animals identified may overestimate the percent of culture-positive animals that could be expected in all non-reactors in a particular herd because a proportion of the non-reactors selected in the study were included due to this high risk profile (i.e., unspecific reactions in previous herd tests and suspected anergy) and therefore do not represent the overall non-reactor population. Similarly, a proportion of non-reactors included in the study with negative bacteriology may have been truly infected but missed due to the limited sensitivity of culture, especially in early stages of infection when bacterial load is low [9]. Still, our study design is well suited for identifying factors associated with non-reactors with an infection detectable by culture, which could indicate a higher bacterial load in their tissues, a more advanced stage of infection and a higher risk of shedding the bacteria, rendering its detection a key factor to control and eventually eradicate infection from a herd. These animals would remain undetected if diagnostic tests are applied according to the protocols, and therefore represent a risk for persistence of bTB infection in the herd.

## Conclusions

Our results demonstrate that among non-reactors older animals and bullfighting cattle should be monitored carefully due to their increased probability of being infected with *M. bovis* or *M. caprae*, although the proportion of T-/C + cattle remaining in the herd after testing decreased when the IFN-γ assay is used as an ancillary test. When both tests are applied in parallel, the probability of finding culture-confirmed infected animals among non-reactors was significantly lower after the third round of testing compared to what is observed after the first round, suggesting that both techniques should be applied simultaneously at least twice in order to assure the reliability of the results in negative animals. Increased surveillance of non-reactors with an increased probability of being false negative could be helpful to avoid bTB persistence, particularly in chronically infected herds.

## Competing interests

The authors declare that they have no competing interests.

## Authors’ contributions

JA and AP performed the statistical analyses and drafted the manuscript. JB, MLC and BR gathered the data from official databases and all performed descriptive analyses. SM, AG, JLS and OM coordinated information assemblage, sample selection and designed the databases used for data collection. MRE and MCM performed laboratory analyses (*in-vivo* diagnosis and bacteriology). LdJ and LD designed the study and coordinated the work. All authors revised critically the manuscript. All authors read and approved the final manuscript.
